# Statin as a Combined Therapy for Advanced-Stage Ovarian Cancer: A Propensity Score Matched Analysis

**DOI:** 10.1155/2016/9125238

**Published:** 2016-11-16

**Authors:** Hong-Yu Chen, Qian Wang, Qiu-Hong Xu, Li Yan, Xue-Feng Gao, Yan-Hong Lu, Li Wang

**Affiliations:** ^1^Department of Gynecology and Obstetrics, Heilongjiang Provincial Hospital, Harbin 150036, China; ^2^School of Computer Science and Technology, Harbin University of Science and Technology, Harbin 150080, China; ^3^Department of Radiology, Heilongjiang Provincial Hospital, Harbin 150036, China; ^4^Department of Pathology, Heilongjiang Provincial Hospital, Harbin 150036, China

## Abstract

*Background*. Despite the great achievements in the treatment of advanced-stage ovarian cancer, it is still a severe condition with an unfavorable 5-year survival rate. Statins have been suggested to reduce the risk of several cancers beyond their cholesterol-lowing effects. However, the prognostic significance of statins in patients with advanced-stage ovarian cancer remains controversial.* Methods*. A retrospective study was performed to evaluate the association between statin intake and overall survival (OS) among patients with advanced-stage ovarian cancer. Patients who underwent cytoreductive surgery followed by courses of intravenous chemotherapy were matched through a propensity score analysis.* Results*. A total of 60 propensity-matched patients were included. Women in statin group showed a similar OS than the nonstatin counterparts (*P* = 0.966), whereas residual tumor was significantly associated with better OS (*P* = 0.013) and was an independent factor that associated with OS (*P* = 0.002, hazard ratio = 5.460, and 95% confidence interval: 1.894 to 15.742) in multivariable analysis.* Conclusions*. Our results suggested that statin usage was not associated with improved OS in patients with advanced-stage ovarian cancer undergoing surgery and chemotherapy. Considering the retrospective nature and the relative small sample size of the study, further prospective studies and random control trials are needed.

## 1. Introduction

Ovarian cancer is the seventh most common cancer and the eighth cause of death from cancer in women [1]. Though the overall survival can be effectively prolonged by management of radical surgery and chemotherapy, about 70% of newly diagnosed patients are advanced-stage ovarian cancer, with a poor five-year survival rate [2].

Cytoreductive surgery and adjuvant chemotherapy are considered as the standard treatment for advanced-stage ovarian cancer. However, many patients experience recurrence and chemotherapy resistance, even though they response well initially. Consequently, the overall 5-year survival rate is about 40% after diagnosis with advanced-stage ovarian cancer [3]. Thus, effective and accessible adjuvant interventions for advanced-stage ovarian cancer are required.

Statin (3-hydroxy-3-methylglutaryl coenzyme, a reductase inhibitor), which is currently used extensively for treatment of hypercholesterolemia and prevention of cardiovascular disease, has been suggested to be associated with a lower risk of ovaries cancer, including breast, colorectal, and ovarian cancer [4]. It also has been reported to exert antiproliferative and proapoptotic effects on several cancer cells [4]. On the one hand, high serum cholesterol may directly lead to tumor progression [5]. On the other hand, emerging evidences supported the involvement of elevated cholesterol in reprogramming of metabolic program which augments the process of tumor development [6]. It has been suggested that statins inhibit cancer development through their inhibition of the mevalonate pathway, which could modify several proteins which is particularly necessary for the function of cancer cells, such as GTPases Ras, Rho, and Rab [3].

However, the effect of statins in the clinical setting for the management of ovarian cancer patients remains unknown [3, 7]. Few studies reported the effect of statin therapy on ovarian cancer survival. Previously, a retrospective study included 128 cases suggested that statins significantly prolonged overall survival (OS) compared with nonstatin users (median OS: 62 versus 46 months, *P* = 0.044) [8]. Similarly, Lavie et al. revealed that taking statins after diagnosis, in patients that had not developed their ovarian cancer while being under statin treatment, could have a better OS (*P* = 0.001) [9]. However, in a recent study that enrolled 442 ovarian cancer patients, improved survival among statin users was not demonstrated except in nonserous papillary epithelial ovarian cancer [10]. Furthermore, in some cancer cell lines, combining statin with carboplatin or paclitaxel revealed antagonistic effects, whereas the combination of taxane and platinum is a standard recommendation for ovarian cancer [3, 11].

Therefore, in the present study, we performed a retrospective study to evaluate the association between statin usage and overall survival (OS) among patients with advanced-stage ovarian cancer (International Federation of Gynecology and Obstetrics (FIGO) stage III or IV) who underwent comprehensive treatment.

## 2. Methods

### 2.1. Study Patients and Following-Up

This retrospective study was approved by the Ethics Committee of the Heilongjiang Provincial Hospital. The study was conducted using the databases of Department of Gynecology and Obstetrics, Heilongjiang Provincial Hospital. All patients diagnosed with ovarian cancers were selected for the study.

Inclusion and exclusion criteria were as follows: (1) patients with FIGO stage III or IV ovarian cancer who received the primary treatment with the combination of cytoreductive surgery and courses of platinum-based intravenous chemotherapy (carboplatin (0.3–0.4 mg/m^2^) or cisplatin (50–120 mg/m^2^) combined with paclitaxe (135–200 mg/m^2^)) between January 1, 2009, and December 31, 2013, at the Department of Gynecology and Obstetrics, Heilongjiang Provincial Hospital, were enrolled. (2) Patients had no prior history of cancer. (3) According to the medical records, the outpatients prescription records, and phone call following-up records, patients who were prescribed statins for a period longer than three months during the observation period (from the diagnosis of ovarian cancer to the date of the latest follow-up or death) were defined as statin users. Finally, 35 patients with statins intake in addition to the routine comprehensive treatment were included.

Following-up was performed by hospital visitation or telephone. All patients were followed up until December 31, 2015. The median follow-up period was 30.3 ± 14.9 months (range: 6.1–57.8 months).

The primary endpoint was overall survivals (OS), which was defined as the duration from the date of the initial treatment to the date of death or last follow-up. Patients alive on December 31, 2015, and patients lost during following-up were defined as censored data.

### 2.2. Statistical Analysis

Considering the selection bias from potential confounders, propensity score matching was performed. The Pamatch2 Macro in Stata version 11.0 (StataCorp LP, College Station, TX, USA) was used for the propensity score matching. The nearest-neighbor matching method was used to perform a 1 : 1 matching as described previously [12]. Briefly, the variables for calculating the propensity score were age at diagnosis, tumor grade, FIGO stage, histological subtype, numbers of chemotherapy cycles after surgery, residual tumor size, and comorbidity for which statins were prescribed (hypercholesterolemia, cardiovascular diseases, or others). Propensity scores ranging from 0 to 1 were generated using binary logistic regression. Distribution of propensity scores was evaluated for the sufficient overlap between two groups to ensure comparability. Baseline characteristics of patients before propensity score matching were listed in Supplementary Table 1 (in Supplementary Material available online at http://dx.doi.org/10.1155/2016/9125238).

All data were analyzed using SPSS version 19.0 for Windows. All statistical tests were two-sided and *P* < 0.05 was defined as significant. Numbers with percentages for categorical variables and median for continuous variables were used to show clinical characteristics. Student's *t*-test or Chi-square test was used to test variables between two groups. The Kaplan-Meier method and the log-rank test were used to perform survival curves. The Cox proportional hazards model, hazard ratio (HR), and 95% confidence interval (95% CI) were used to adjust the HRs with the other factors based on clinical considerations and identified independent factor associated with OS.

## 3. Results

A total of 35 statin users were identified in all patients with FIGO stage III or IV ovarian cancer who underwent the comprehensive treatment during the study period. After propensity matching, 30 matched pairs (60 women) were included in the study. Most of women (21 patients, 70%) were prescribed statins for the treatment of hypercholesterolemia, and 10 patients (33.3%) were prescribed statins for the treatment of cardiovascular diseases. The majority of the women were prescribed a daily dose of 10–20 mg. The clinical characteristics of patients are shown in Table 1, and between the two groups, no significant differences were found.

**Figure 1:**
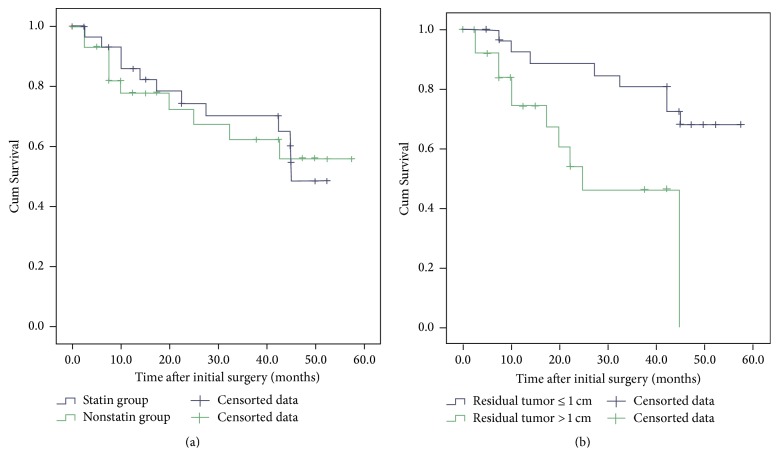
Kaplan-Meier curves showing the survival differences between the two groups: (a) statin users versus nonstatin users and (b) residual tumor < 1 cm versus residual tumor > 1 cm after cytoreductive surgery.

The median follow-up was 30.3 ± 14.9 months (range from 6.1 to 57.8 months). During the study period, there were a total of 22 deaths observed: 10 in statin usage group and 12 in nonstatin group. Six deaths were ovarian cancer-related in statin users and nine in nonstatin users. In all the 60 patients, the 1-, 2-, 3-, and 4-year cumulative survival rates were 82%, 74%, 67%, and 53%, respectively. In statins usage women, the 1-, 2-, 3-, and 4-year cumulative survival rates were 78%, 74%, 63%, and 57%, respectively, while in nonstatin group, they were 86%, 75%, 71%, and 51%, respectively. The overall survival had no significant difference between the statin group and the matched nonstatin group (*P* = 0.966, Figure 1(a)) during the study period. In addition, patients with FIGO III had a 3-year cumulative survival rates of 69%, whereas FIGO IV showed 64%. The 3-year cumulative survival rates in patients with tumor grades 1-2, tumor grade 3, epithelial subtype, and nonepithelial subtype were 81%, 63%, 89%, and 63%, respectively.

In univariable analysis, small residual tumor size (≤1 cm) was revealed to be associated with better OS (*P* = 0.013, Figure 1(b), Table 2). In addition, further multivariable analysis using Cox hazard regression showed residual tumor was an independent factor that associated with OS rather than other factors (*P* = 0.002, HR = 5.460, and 95% CI: 1.894 to 15.742, Table 2).

## 4. Discussion

Despite the great achievements in the treatment of advanced ovarian cancer, it is still a severe condition with an unfavorable 5-year survival rate. Therefore, a series of new treatments and drugs are undergoing clinical evaluation, including statins [3]. Statins have been found to have additional anticancer potential through inducting cancer cells apoptosis and inhibiting cancer cells proliferation, invasion, and metastasis in preclinical studies [13]. And some clinical studies have suggested the association between statins usage and better OS in patients with cancer [14].

However, in the present retrospective study, we demonstrated that statin usage (10–20 mg daily) did not associate with prolonged OS in women with FIGO stage III or IV ovarian cancer who underwent cytoreductive surgery followed by chemotherapy. Consistent with our finding, a recent study found that the risk for ovarian cancer recurrence or disease-specific death was not significantly lower in patients with hyperlipidemia taking statins when compared to patients without hyperlipidemia or to those hyperlipidemics who did not take statins [10]. Of note, this study found a trend toward better prognosis among statin users with nonpapillary serous histological subtypes [10]. Thus, a possible explanation might be that the protective effects of statins could be limited to specific subtypes of ovarian cancer, since ovarian cancer is heterogeneous and different histological subtypes exhibit different clinical profiles [15].

Other potential explanations for the negative association between statin usage and ovarian cancer OS may be that the dose and duration of statins intake would influence their anticancer effects. For example, a recent nested case-control study suggested a dose and duration effect of statins in esophageal adenocarcinoma development, as malignant cases had dispensed prescriptions with significantly lower cumulative statin dose (<15 g, 17.4% malignant cases versus 25.9% controls; 15–30 g, 10% versus 9.8%; 30–60 g, 4.2% versus 6.3%; >60 g, 3.2% versus 4.9%) and shorter statin take duration (<6 months, 10.9% malignant cases versus 13% controls; 6–18 months, 10% versus 17.1%; >18 months, 19.3% versus 23.9%) than Barrett's esophagus controls [16].* In vitro* study also revealed that the concentration of statin required in ovarian cancer cell lines was significantly higher than that achieved in plasma when patients receive the dose of statin (about 40 mg daily) commonly used to treat hypercholesterolemia [3]. In addition, it has been suggested that statin usage before diagnosis of colon cancer is associated with improved OS, whereas no such survival benefit was indicated for patients taking statins after colon cancer diagnosis [17].

Among all the improvements in the treatment of this disease, the role of optimal cytoreduction surgery particularly stands out. Even in FIGO stage IV ovarian cancer, the prolonged OS were obtained by complete cytoreduction surgery, which range from 48 to 72 months [18]. Consistent with this, our study revealed the residual tumor size after cytoreduction surgery is an independent factor that associated with OS in patients with advanced-stage ovarian cancer.

Our study has several limitations. First, retrospective studies have inherent limitations. For example, only well recorded data in database were included in the study and we cannot rule out the possibility of recall bias. Second, women in statin usage group were prescribed statins for treatment of hypercholesterolemia or prevention of other diseases, especially acute coronary syndrome. Though propensity score matching analysis was performed, it is not possible to eliminate all the influence of these comorbidities on patients overall survival. What is more, due to the small sample size and the relatively short mean follow-up time, the methodological limitations could not be ruled out.

## 5. Conclusion

Our result demonstrated that, in patients with advanced-stage ovarian cancer undergoing surgery and chemotherapy, statin usage was not associated with better overall survival. Considering the retrospective nature and the relative small sample size of our study, the results must be interpreted with caution. Further prospective studies and random control trials are needed.

## Supplementary Material

The baseline characteristics of patients before propensity score matching and survival curves of certain patients were listed in Supplementary Material.

## Figures and Tables

**Table 1:** Baseline characteristics of statin users (*n* = 30) and matched nonstatin users (*n* = 30).

Characteristics	Statin users number (%)	Matched nonstatin users number (%)	*P*
Age	68.48 ± 7.62	68.16 ± 7.50	
<65 years	13 (43.3)	12 (40)	1
≥ 65	17 (56.7)	18 (60)	
FIGO stage			
III	17 (56.7)	19 (63.3)	0.792
IV	13 (43.3)	11 (36.7)	
Tumor grade			
G1-G2	9 (30)	4 (13.3)	0.209
G3	21 (70)	26 (86.7)	
Histological subtype			
Epithelial	4 (13.3)	6 (20)	0.731
Nonepithelial	26 (93.3)	24 (80)	
Cytoreductive surgery			
Residual tumor > 1 cm	13 (43.3)	14 (46.7)	0.888
Residual tumor ≤ 1 cm	14 (46.7)	14 (46.7)	
Not recorded	3 (10)	2 (6.7)	
Cycles of chemotherapy			
≥6 cycles	27 (90)	25 (83.3)	0.331
Not recorded	2 (6.7)	1 (3.3)	
Comorbidities			
Hypercholesterolemia	21 (70)	21 (70)	1
Cardiovascular diseases	10 (33.3)	10 (33.3)	1

**Table 2:** Univariable and multivariable analysis: risk of mortality.

Characteristics	Univariable analysis *P*	Multivariable analysis *P*	HR (95% CI)
Age (≥65/<65 years)	0.535	0.734	1.176 (0.461–2.999)
FIGO stage (IV/III)	0.958	0.359	1.614 (0.581–4.485)
Tumor grade (G3/G1-2)	0.254	0.193	2.558 (0.622–10.513)
Histological subtype (Epithelial/nonepithelial)	0.653	0.752	1.250 (0.315–4.964)
Cytoreductive surgery (Residual tumor > 1/≤1 cm)	0.013	0.002	5.460 (1.894–15.742)
Statin (nonusage/usage)	0.966	0.255	1.764 (0.664–4.682)

## References

[1] Torre L. A., Bray F., Siegel R. L., Ferlay J., Lortet-Tieulent J., Jemal A. (2015). Global cancer statistics, 2012. *CA: A Cancer Journal for Clinicians*.

[2] Pranjol M. Z., Gutowski N., Hannemann M., Whatmore J. (2015). The potential role of the proteases cathepsin D and cathepsin L in the progression and metastasis of epithelial ovarian cancer. *Biomolecules*.

[3] Robinson E., Fisher N., Stamelos V., Redman C., Richardson A. (2014). New strategies for the treatment of ovarian cancer. *Biochemical Society Transactions*.

[4] Stryjkowska-Góra A., Karczmarek-Borowska B., Góra T., Krawczak K. (2015). Statins and cancers. *Contemporary Oncology*.

[5] Allott E. H., Howard L. E., Cooperberg M. R. (2014). Serum lipid profile and risk of prostate cancer recurrence: results from the SEARCH database. *Cancer Epidemiology Biomarkers and Prevention*.

[6] Mandal C. C., Rahman M. M. (2014). Targeting intracellular cholesterol is a novel therapeutic strategy for cancer treatment. *Journal of Cancer Science & Therapy*.

[7] Jacobs E. J., Newton C. C., Thun M. J., Gapstur S. M. (2011). Long-term use of cholesterol-lowering drugs and cancer incidence in a large United States cohort. *Cancer Research*.

[8] Elmore R. G., Ioffe Y., Scoles D. R., Karlan B. Y., Li A. J. (2008). Impact of statin therapy on survival in epithelial ovarian cancer. *Gynecologic Oncology*.

[9] Lavie O., Pinchev M., Rennert H. S., Segev Y., Rennert G. (2013). The effect of statins on risk and survival of gynecological malignancies. *Gynecologic Oncology*.

[10] Habis M., Wroblewski K., Bradaric M. (2014). Statin therapy is associated with improved survival in patients with non-serous-papillary epithelial ovarian cancer: a retrospective cohort analysis. *PLoS ONE*.

[11] Troso-Sandoval T. A., Lichtman S. M. (2015). Chemotherapy of ovarian cancer in elderly patients. *Cancer Biology and Medicine*.

[12] Ju M.-J., Tu G.-W., Han Y. (2013). Effect of admission time on mortality in an intensive care unit in Mainland China: a propensity score matching analysis. *Critical Care*.

[13] Park Y. H., Jung H. H., Ahn J. S., Im Y.-H. (2013). Statin induces inhibition of triple negative breast cancer (TNBC) cells via PI3K pathway. *Biochemical and Biophysical Research Communications*.

[14] Nimako G. K., Wintrob Z. A., Sulik D. A., Donato J. L., Ceacareanu A. C. (2016). Synergistic benefit of statin and metformin in gastrointestinal malignancies. *Journal of Pharmacy Practice*.

[15] Jayson G. C., Kohn E. C., Kitchener H. C., Ledermann J. A. (2014). Ovarian cancer. *The Lancet*.

[16] Nguyen T., Duan Z., Naik A. D., Kramer J. R., El-Serag H. B. (2015). Statin use reduces risk of esophageal adenocarcinoma in US veterans with Barrett's Esophagus: A Nested Case-Control Study. *Gastroenterology*.

[17] Ling Y., Yang L., Huang H. (2015). Prognostic significance of statin use in colorectal cancer: a systematic review and meta-analysis. *Medicine*.

[18] Bacalbasa N., Balescu I., Dima S. (2015). Initial incomplete surgery modifies prognosis in advanced ovarian cancer regardless of subsequent management. *Anticancer Research*.

