# Supplementary material for: Statin as a Combined Therapy for Advanced-Stage Ovarian Cancer: A Propensity Score Matched Analysis
Source: Biomed Res Int. 2016 Nov 16;2016:9125238. doi: 10.1155/2016/9125238 (PMC5128698; doi:10.1155/2016/9125238)

Supplementary table1. Baseline characteristics of patients before propensity score matching

| Characteristics | statin users  number (n=35) ^a^ | non-statin users  number (n=201) ^b^ | *P* |
| --- | --- | --- | --- |
| Age |  |  |  |
| ＜65 years | 14 | 122 | 0.026 |
| ≥65 | 21 | 79 |  |
| FIGO stage |  |  |  |
| Ⅲ | 17 | 140 | 0.020 |
| Ⅳ | 18 | 61 |  |
| Tumor grade |  |  |  |
| G1-G2 | 9 | 45 | 0.666 |
| G3 | 26 | 156 |  |
| Histological subtype |  |  |  |
| Epithelial | 4 | 36 | 0.466 |
| Non-epithelial | 31 | 165 |  |
| Cytoreductive surgery |  |  |  |
| Residual Tumor＞1 cm | 17 | 90 | 0.442 |
| Residual Tumor≤1 cm | 14 | 103 |  |
| Not recorded | 4 | 8 |  |
| Cycles of Chemotherapy |  |  |  |
| ≥6 cycles | 28 | 150 | 0.671 |
| Not recorded | 7 | 51 |  |
| Comorbidities |  |  |  |
| Hypercholesterolemia | 23 | 32 | 0.022 |
| Cardiovascular diseases | 10 | 18 |  |
| Others | 2 | 151 |  |

^a^Patients fulfilled the inclusion and exclusion criteria listed in the Method section.

^b^Patients without statin usage who fulfilled the first and the second inclusion criteria and the exclusion criteria listed in the Method section.

Supplementary figure 1. Survival curves in patients in two groups (FIGO Ⅲ vs. Ⅳ): (A) in all 60 patients (*P*=0.958); (B) in patients with statin usage (*P*=0.308); (C) in patients without statin usage (*P*=0.334).


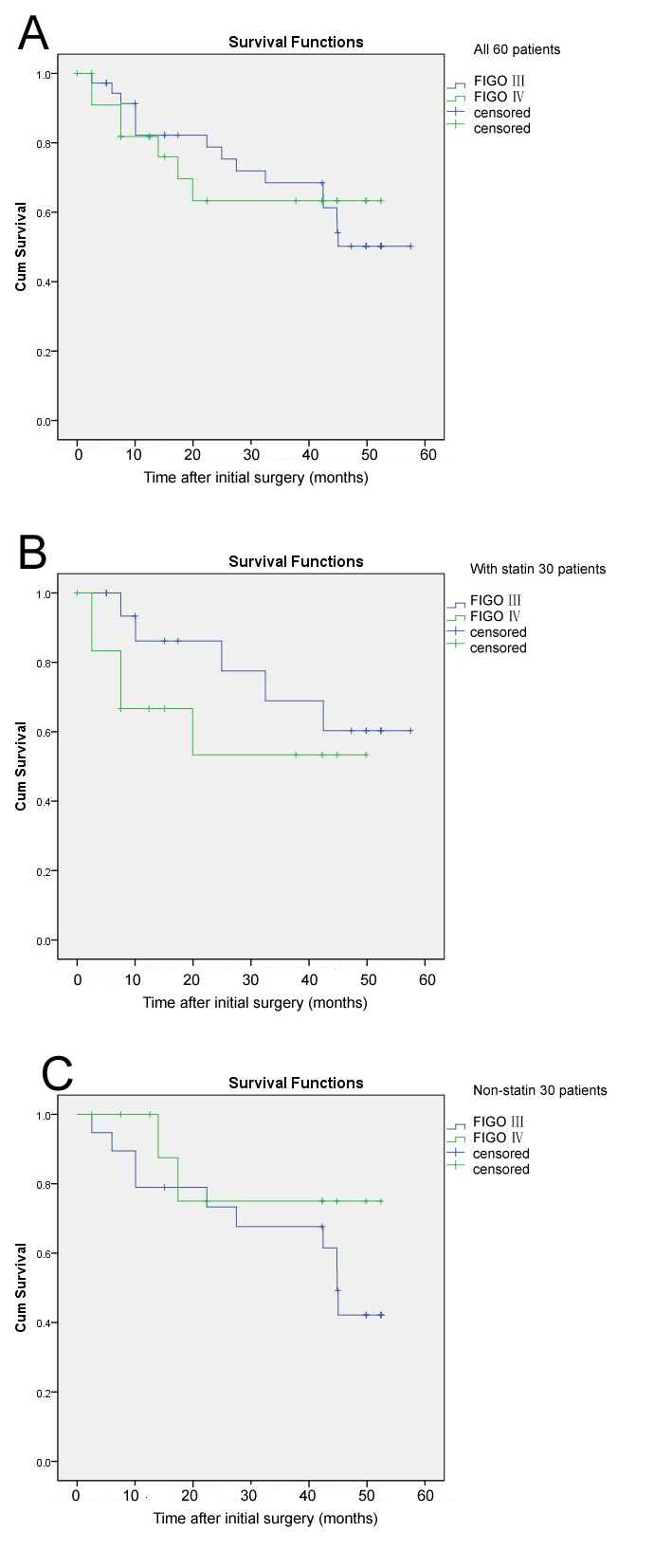


Supplementary figure 2. Survival curves in patients in two groups (Tumor grade G1-2 vs. G3): (A) in all 60 patients (*P*=0.254); (B) in patients with statin usage (*P*=0.253); (C) in patients without statin usage (*P*=0.715).


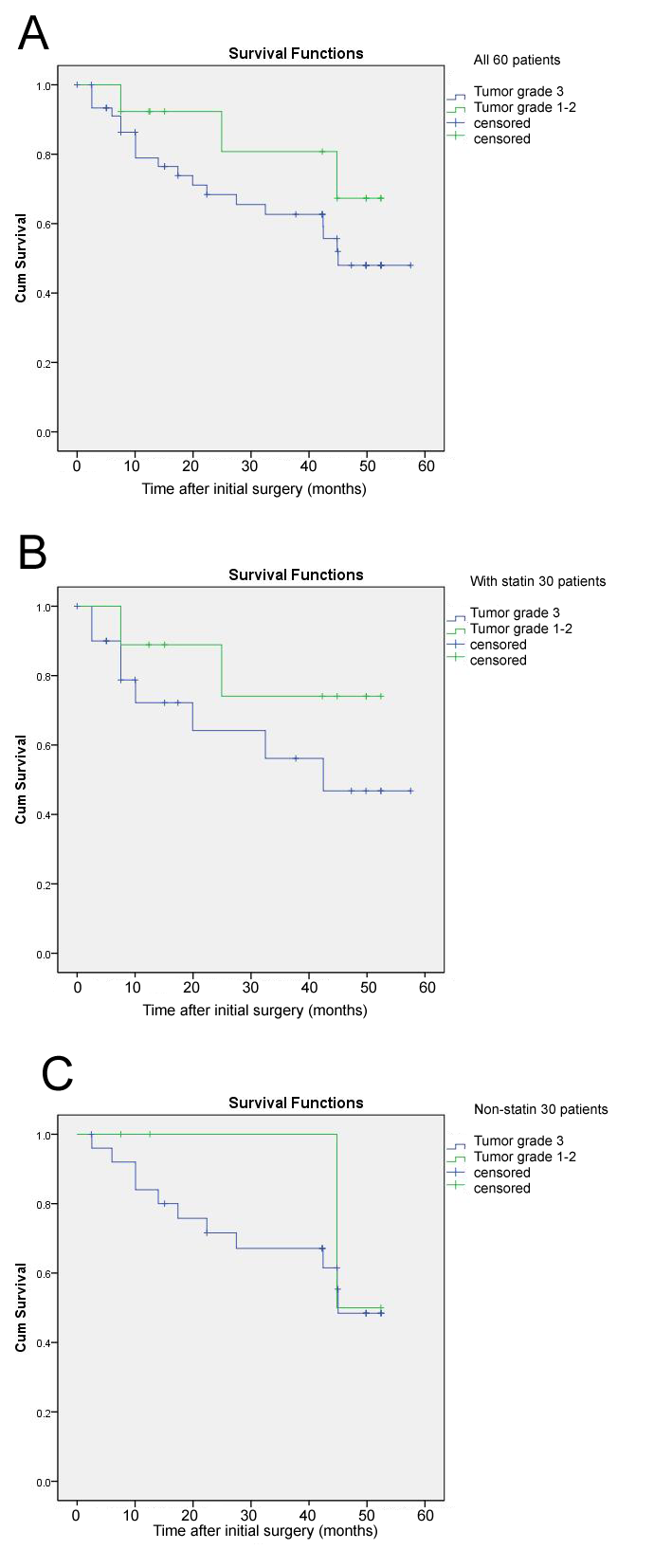


Supplementary figure 3. Survival curves in patients in two groups (Epithelial vs. Non-epithelial): (A) in all 60 patients (*P*=0.653); (B) in patients with statin usage (*P*=0.617); (C) in patients without statin usage (*P*=0.919).


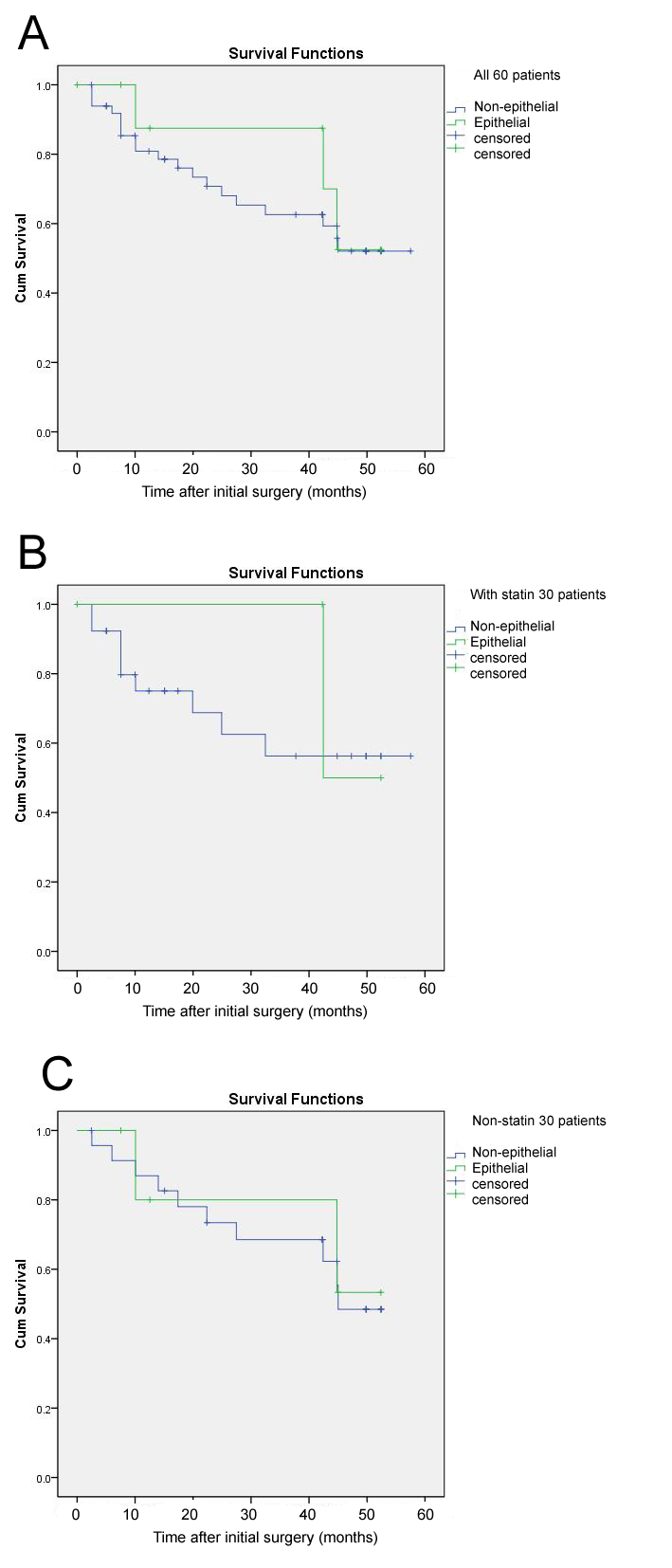

Supplement: Supplementary file 1 — The baseline characteristics of patients before propensity score matching and survival curves of certain patients were listed in Supplementary Material. [file 9125238.f1.docx]
